# NPR1 Protein Regulates Pathogenic and Symbiotic Interactions between *Rhizobium* and Legumes and Non-Legumes

**DOI:** 10.1371/journal.pone.0008399

**Published:** 2009-12-21

**Authors:** Smadar Peleg-Grossman, Yael Golani, Yuval Kaye, Naomi Melamed-Book, Alex Levine

**Affiliations:** Department of Plant and Environmental Sciences, The Hebrew University of Jerusalem, Jerusalem, Israel; University of Heidelberg, Germany

## Abstract

**Background:**

Legumes are unique in their ability to establish symbiotic interaction with rhizobacteria from *Rhizobium* genus, which provide them with available nitrogen. Nodulation factors (NFs) produced by *Rhizobium* initiate legume root hair deformation and curling that entrap the bacteria, and allow it to grow inside the plant. In contrast, legumes and non-legumes activate defense responses when inoculated with pathogenic bacteria. One major defense pathway is mediated by salicylic acid (SA). SA is sensed and transduced to downstream defense components by a redox-regulated protein called NPR1.

**Methodology/Principal Findings:**

We used *Arabidopsis* mutants in SA defense pathway to test the role of NPR1 in symbiotic interactions. Inoculation of *Sinorhizobium meliloti* or purified NF on *Medicago truncatula* or *nim1/npr1 A. thaliana* mutants induced root hair deformation and transcription of early and late nodulins. Application of *S. meliloti* or NF on *M. truncatula* or *A. thaliana* roots also induced a strong oxidative burst that lasted much longer than in plants inoculated with pathogenic or mutualistic bacteria. Transient overexpression of *NPR1* in *M. truncatula* suppressed root hair curling, while inhibition of *NPR1* expression by RNAi accelerated curling.

**Conclusions/Significance:**

We show that, while NPR1 has a positive effect on pathogen resistance, it has a negative effect on symbiotic interactions, by inhibiting root hair deformation and *nodulin* expression. Our results also show that basic plant responses to *Rhizobium* inoculation are conserved in legumes and non-legumes.

## Introduction

Plants continually interact with soil micro-organisms that are broadly divided into pathogenic, saprophytic or symbiotic. While the pathogenic and saprophytic interactions are common to all plant species, symbiosis with the nitrogen-fixing rhizobacteria is a relatively recent evolutionary development that is restricted to plants of the legumes family. Legumes are unique in their ability to establish symbiotic interaction with rhizobacteria from the *Rhizobium* genus, which provide plants with a source of available nitrogen. Symbiosis is regulated by complex mutual interactions between the organisms. Symbiosis between legumes and *Rhizobium* is initiated by specific nodulation (Nod) factors (NFs) that are secreted into the soil by the bacteria. In response to compatible NFs the legume root hairs begin to curl, entrapping the bacteria. The cell wall within the curl undergoes a local hydrolysis, allowing bacteria to enter the root hair and form an intracellular infection thread from the curled region to the root cortex [1]. The root cells in the cortex undergo reprogramming and begin to divide rapidly, giving rise to a nodule primordium, a specific plant organ that provides favorable environment for nitrogen fixation by *Rhizobium* [2].

In contrast to symbiotic interaction with rhizobacteria, plants mount a defense response when challenged with pathogenic bacteria. Several signaling pathways that mediate local and systemic plant responses to pathogens have been identified. One of the major signaling pathways induced during pathogenic interactions, including local and systemic defense responses to pathogens is mediated by salicylic acid (SA); for reviews: [3], [4]. SA is sensed and transduced by NPR1 protein, which is a redox-sensitive protein that contains several ankyrin repeats and has limited homology to IκBα [5]. During pathogenesis response, the challenged cells undergo an oxidative burst followed by reduction of two conserved cysteines in the NPR1, leading to its monomerization and nuclear localization. In its reduced form, the NPR1 protein interacts with bZIP transcription factors of the TGA/OBF family, and activates the SA-responsive element in the promoters of defense genes, such as pathogenesis related protein, PR1 [6]. Increased production of SA, or *NPR1* overexpression cause enhanced disease resistance (*edr1*) phenotype in heterologous plant species, suggesting an evolutionary conserved SA-mediated signaling pathway in different plants [7]. Moreover, mutations that block SA perception and signaling, such as *nim1*/*npr1*, as well as mutations that reduce SA production (*pad4* or *eds1*) suppressed the *edr* phenotype in all of the *edr* mutants [8]. Furthermore, the *edr* phenotype was also suppressed by expression of SA hydroxylase *NahG* transgene, which converts SA to catechol, resulting in rapid SA decomposition [9].

Compared to the pathogenic interactions only a few studies addressed the involvement of SA in symbiotic interactions. SA measurements in *M. truncatula* during the first stages of symbiotic interaction with *Rhizobium* showed a reduction in the amount of SA [10]. Moreover, reduction of endogenous SA levels in *M. truncatula* by the *NahG* transgene resulted in increased rhizobial infection and nodulation. Furthermore, inoculation of incompatible strains of *S. meliloti* on alfalfa (*Medicago sativa*) roots led to accumulation of SA, and exogenous application of SA to alfalfa plants inhibited nodule formation [10]. Aborted infections were also shown to be accompanied by an HR-like defense response, including necrosis and accumulation of PR proteins, suggesting activation of plant defense responses in aborted *S. meliloti* infection threads [11], [12]. Exogenous SA addition was also shown to inhibit indeterminate nodulation (e.g., in vetch, with a persistent meristem), but not in determinate nodulation (e.g., in *Lotus japonicus* with no persistent meristem) [13]. Interestingly, ROS production has been recently shown to occur not only in pathogenic interactions but also during symbiotic interactions [14]. Physiological concentrations of SA were also shown to markedly increase defense gene induction and H_2_O_2_ accumulation in soybean infected with avirulent pathogens [15]. Thus, redox and SA signaling, both may have direct effects during symbiotic, as well as pathogenic interaction.

Here, we show that SA and NPR1 negatively affect the symbiotic interactions between *M. truncatula* and *Rhizobium*. We also show that *npr1* mutants in non-legume *A. thaliana* respond to *S. meliloti* by activating root hair deformation and induction of early and late *nodulin* genes. Interestingly, both *M. truncatula* and *npr1* mutant *A. thaliana* responded with an extremely strong oxidative burst to *S. meliloti* inoculation, which lasted beyond the restoration of redox after inoculation of *Pseudomonas putida* or *Pseudomonas syringae*.

## Results/Discussion

### The Effect of Salicylic Acid on Root Hair Deformation Following *Sinorhizobium meliloti* Inoculation

Salicylic acid is a major regulator of plant defenses to pathogenic microorganisms, and was shown to adversely affect plant symbiotic interactions [12]. We analyzed the early steps in legume-*Rhizobium* interaction, involving root hair deformation that precedes hair curling in legumes. Root hair deformation is one of the first steps in interaction with compatible rhizobacteria [16]. To examine the effect of SA on *Rhizobium*-induced root hair deformation we first pretreated *M. truncatula* seedlings with SA prior to inoculation of *S. meliloti*, which resulted in the inhibition of root hair deformation (compare Fig. S1F with S1C). SA pre-treatment also inhibited the root hair deformation by NF (compare Fig. S1E with S1B).

To analyze the SA-mediated signaling during symbiotic interactions in *M. truncatula*, we tested the expression of the *alpha*-*Dioxygenase* (*α*-*Dox*) gene that is regulated by SA in tomato, tobacco and *Arabidopsis thaliana* [17], [18], [19], [20]. The *alpha*-*Dox* gene expression in *M. truncatula* roots was reduced during the first day after *S. meliloti* inoculation (Fig. S1G), which is in agreement with the reduced amount of SA seen in *Medicago sativa* during the first stage of rhizobial infection [10].

To explore if the root hair deformation is a typical legume response to compatible *Rhizobium* species, or a general plant response to rhizobacteria, we examined the root hair responses in a non-legume *Arabidopsis thaliana*. Seedlings were inoculated with *S. meliloti*, or with mutualistic *P. putida* or with pathogenic *P. syringae* bacteria [21]. We chose several mutants that are compromised in pathogenesis responses to avoid possible activation of pathogenesis-associated hypersensitive reaction (HR) that may obscure other physiological responses. We focused on the major pathogen resistance pathway that is mediated by SA: *dnd1* (defense no death), which are mutated in cyclic nucleotide-gated ion channel [22], *ndr1-1* (nonrace-specific disease resistance), which encodes a plasma membrane protein with unknown function [23], and *nim1* (noninducible immunity) [24], also called *npr1* (nonexpresser of PR genes) [25]. The list of mutants and their putative signaling pathways is summarized in Table S1.

**Figure 1:**
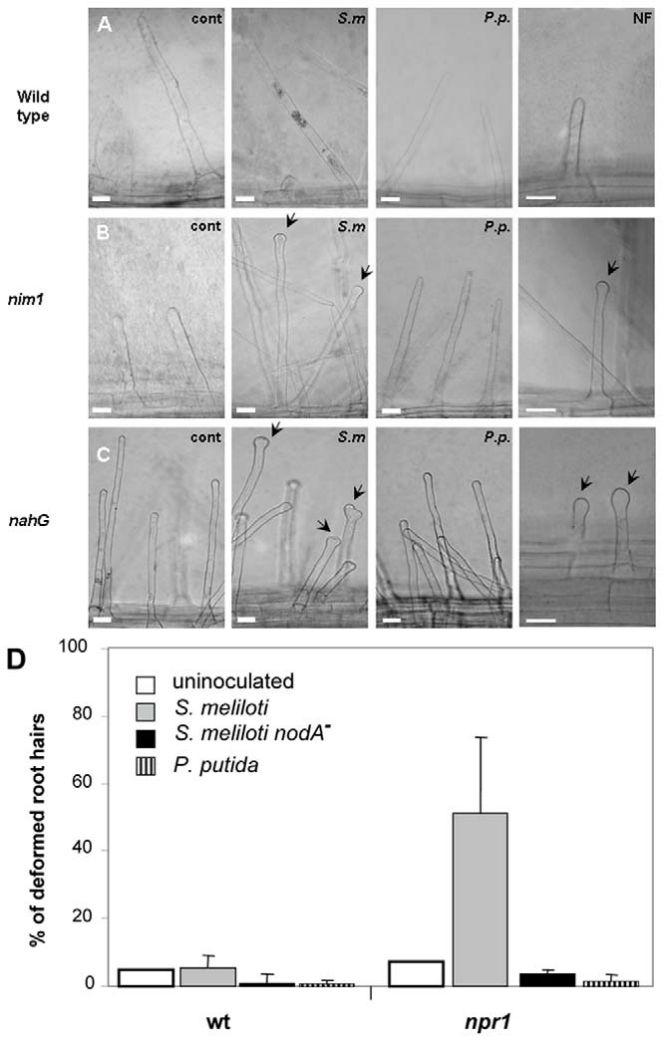
Induction of root hair deformation and attachment of *S. meliloti* to *A. thaliana* hairs. (A–C) Wild-type (A), *nim1/npr1* mutant (B) and *NahG* transformed (C) *A. thaliana* seedlings were grown on nitrogen poor medium (1/60 strength MS) for eight days. Plants were left untreated (A–C, cont), inoculated with *S. meliloti* in zone 1 region (lower-mid root) (*S.m*), inoculated with *P. putida* (*P.p*), inoculated with *S. meliloti nodA* [58] or *nodH* mutants (not shown) or treated with purified nod factors (NF). Roots were photographed 4 days after inoculation under bright light. Bar = 25 µm. Arrows point to deformed root tips. (D) Quantization of root hair deformation response in *A. thaliana* plants inoculated with *S. meliloti* producing intact nod factor (grey) or *nodA* ^−^ mutant (black), *P. putida* (black stipes), or left uninoculated (white). One hundred root hairs from 10 seedlings of wild-type and *nim1/npr1* mutants in zone 1 were scored in each treatment. Error bars represent SD. The experiment was repeated at least 3 times with similar results.

*A. thaliana* seeds were germinated on nitrogen poor medium, and inoculated in the root elongation zone with *S. meliloti* eight days later. No difference in root hair behavior was seen in *dnd1* (Fig. S2), or in *ndr1* mutants, that do not show HR (data not shown) [22], [23]. However, a very strong root hair deformation and even hair bending were detected in the *nim1/npr1* seedlings (Fig. 1B, and Fig. S2). Root hair deformation was specific for *S. meliloti* that produced intact NFs, as no such effect was seen in plants inoculated with mutant *S. meliloti*, in the nod factor genes, *nodA* (Fig. 1D), or *nodH* (data not shown). No deformation was seen also in plants inoculated with *P. putida* (Fig. 1A-C, *P* *.p*). Interestingly, quantitative analysis of *A. thaliana nim1/npr1* mutants showed a similar percentage of deformed root hairs (Fig. 1D), as in *M. truncatula dmi2* mutants after rough NF application/treatment [26]. Importantly, no root hair deformation was seen in plants inoculated with *Pseudomonas syringae* or *P. putida*, or following medium refreshment, as in the case described by Esseling et al. It should be noted that in our experiments the seedlings were left intact in the Petri dish throughout the whole experiment, and were not manipulated before or during the microscopic observation. Thus, in our case the root hair phenotype is not related to touch response during the experimental handling [26].

In addition, we inoculated SA-deficient *A. thaliana* with *S. meliloti* that were transformed with *NahG* [27]. The *NahG*-transformats mimicked the root hair deformation of *nim1/npr1* (Fig. 1C). Also, in the case of *NahG* plants, the effect was a specific response to *S. meliloti*, since no deformation was seen upon inoculation of *P. putida* or *P. syringae* (not shown).

To substantiate the NF-dependent early signaling in non-legume *A. thaliana*, we applied purified nod factor from the *S. meliloti* strain used above to seedlings' roots, without the bacteria. The NF treatment induced root hair deformation exclusively in the SA-insensitive *nim1/npr1* mutants, or in SA-deficient *NahG* transformants, in agreement with data observed in plants treated with intact *S. meliloti* (Fig. 1, NF). It should be noted that although root hair deformation in *A. thaliana* was significant, we did not observe branching that was seen in *M. truncatula* (Fig. S3).

**Figure 2:**
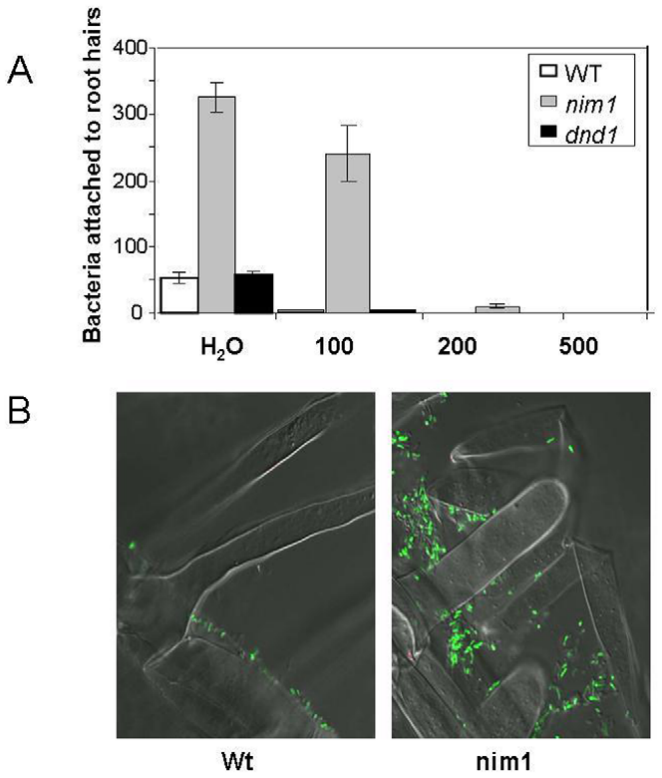
Attachment of *Rhizobium* to *A. thaliana* root hairs. (A) Wild-type *nim1/npr1* and *dnd1* mutants were inoculated with *S. meliloti*. Four days after inoculation plant roots were rinsed with phosphate buffer, as described by [31], followed by buffer supplemented with 100, 200, or 500 mM NaCl. *S. meliloti* in 2500 µm^2^ area in the vicinity of *A. thaliana* root hairs were vewed at 600X magnification with Olympus IX70 microscope and quantified from six roots of each treatment. Wild-type (white), *nim1/npr1* (grey) or *dnd1* (black). The *ndr1* mutants showed similar results to *dnd1*, and therefore were not included. Error bars represent SD. (B) Confocal image of GFP-expressing *S. meliloti* bacteria in the vicinity of wild-type and *nim1/npr1* root hairs after washing with 150 mM NaCl as described in (A).

The attachment of *S. meliloti* to legume root hairs involves a specific activation of a plant-dependent process, which requires more than just inherent adhesiveness of bacteria to plant cell walls [28], [29]. The attachment process involves secretion of specific glycoprotein lectin-polysaccharides by the host symbiont, which induces formation of biofilm in zone 1 of legume roots [29], [30]. We used GFP-labeled *S. meliloti* to observe the bacteria plant interaction. Strong adherence of *S. meliloti* to the *A. thaliana* root hairs was seen in *nim1/npr1* mutants, but not in wild-type or in *dnd1*, or in *ndr1* roots (Fig. 2A and data not shown). The bacteria remained attached to the *nim1/npr1* roots even after extensive washing (Fig. 2B), as described by [31]. Moreover, increasing the washing stringency by addition of 100 mM NaCl to the wash medium almost completely removed the bacteria from wild type and from *dnd1* mutants, but not from the *nim1/npr1* seedlings. Substantial amount of attached *S. meliloti* in the *nim1/npr1* mutants were observed even after further wash with 200 mM NaCl, which completely removed all bacteria from the wild-type roots (Fig. 2A). These results suggest that the attachment of *S. meliloti* to its host is regulated by SA-dependent signaling in the host.

### Induction of *nodulin* Gene Expression in Arabidopsis following *Rhizobium* Inoculation

**Figure 3:**
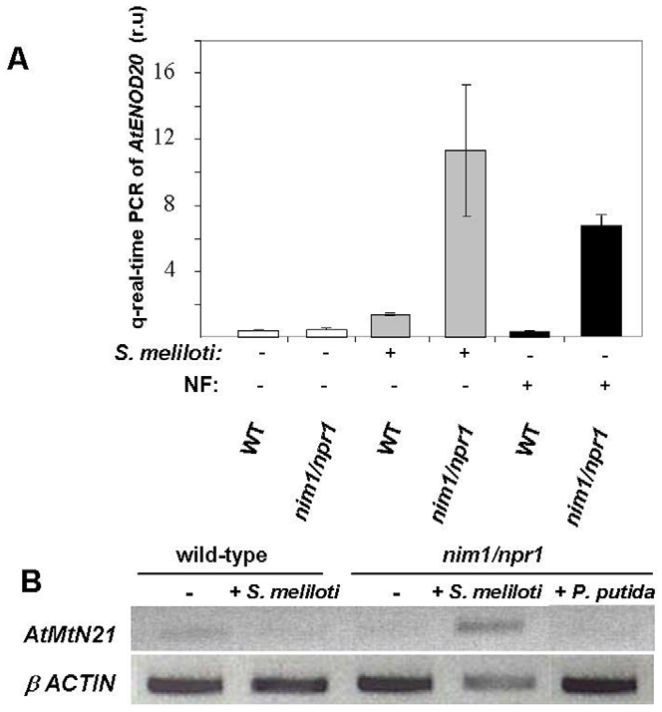
Gene expression analysis of early and late *M. truncatula* nodulin gene orthologs in *A. thaliana* roots. (A) Roots of ten days old wild-type or *nim1/npr1* seedlings grown on nitrogen poor medium were inoculated in zone 1 with either *S. meliloti* or treated with nod factor (NF). The expression of *Arabidopsis ENOD20* homolog (*AtENOD20*, At5g57920) was tested two days after inoculation, using quantitative Real-time RT-PCR. The results show the mean of 3 independent repeats for each treatment. Error bars represent SD. (B) Roots of ten days old wild-type and *nim1/npr1* seedlings grown on nitrogen poor medium were inoculated with *S. meliloti* or with *P.putida.* Late nodulin, (*AtMtN21*, At5g07050) was assayed after 7 days. Plant roots were frozen in liquid nitrogen and gene expression was analyzed by semiquantitative RT-PCR. The RNA samples were normalized according to *actin*-2 gene expression. Experiments were repeated 3 times with very similar results.

The symbiotic interaction between legumes and *Rhizobium* is characterized by induction of nodulin gene expression. Nodulins are divided according to their expression time into early (called ENODs) that act in accommodating the rhizobial bacteria, and late nodulins that are thought to be involved in the nodule functioning. *ENODs* are induced within one or few days after inoculation, while late nodulins take several days [32]. Genomic sequencing has identified nodulin homologs in Arabidopsis and other non-legume genomes of higher plants [33]. The homologs of related gene families in *A. thaliana* are shown in Fig. S4 and Fig. S5. We analyzed the expression of two *ENOD* homologs, representing early (*AtENOD20*, At5g57920) and a late (*AtMtN21*, At5g07050) nodulins, that are expressed 2–5 and 7 days post infection, respectively, in *M. truncatula* [34], [35]. The *A. thaliana ENOD20* homolog, At5g57920, also called early nodulin-like protein in the Arabidopsis TAIR database, shares 38% identity and 56% similarity with the *M. truncatula* protein, while the At5g07050, also called nodulin-related protein, contains 64% identity and 78% similarity. Both genes were induced by the inoculation of *S. meliloti* in the *nim1/npr1* background, but not in wild-type plants (Fig. 3B). The induction was specific for inoculation of *S. meliloti*, but not of *P. putida* bacteria. Moreover, we tested the induction of At5g57920 following NF treatment. Strong induction was detected only in the *nim1/npr1* background (Fig. 3A).

### Induction of Oxidative Burst in *A. thaliana* and *M. truncatula* Plants Inoculated with *S. meliloti* or with *P. putida* or with *P. syringae* Bacteria

A major hallmark of plant interaction with microorganisms, which is particularly characteristic of pathogen attack is generation of reactive oxygen species (ROS), which leads to hypersensitive cell death [36], [37]. Recently, however, ROS production was also observed in symbiotic interactions in *M. truncatula* roots inoculated with *S. meliloti* [38], or treated with compatible Nod Factor [39]. Moreover, oxidative burst was shown to play an important role in the formation of *S. meliloti* infection threads [40].

**Figure 4:**
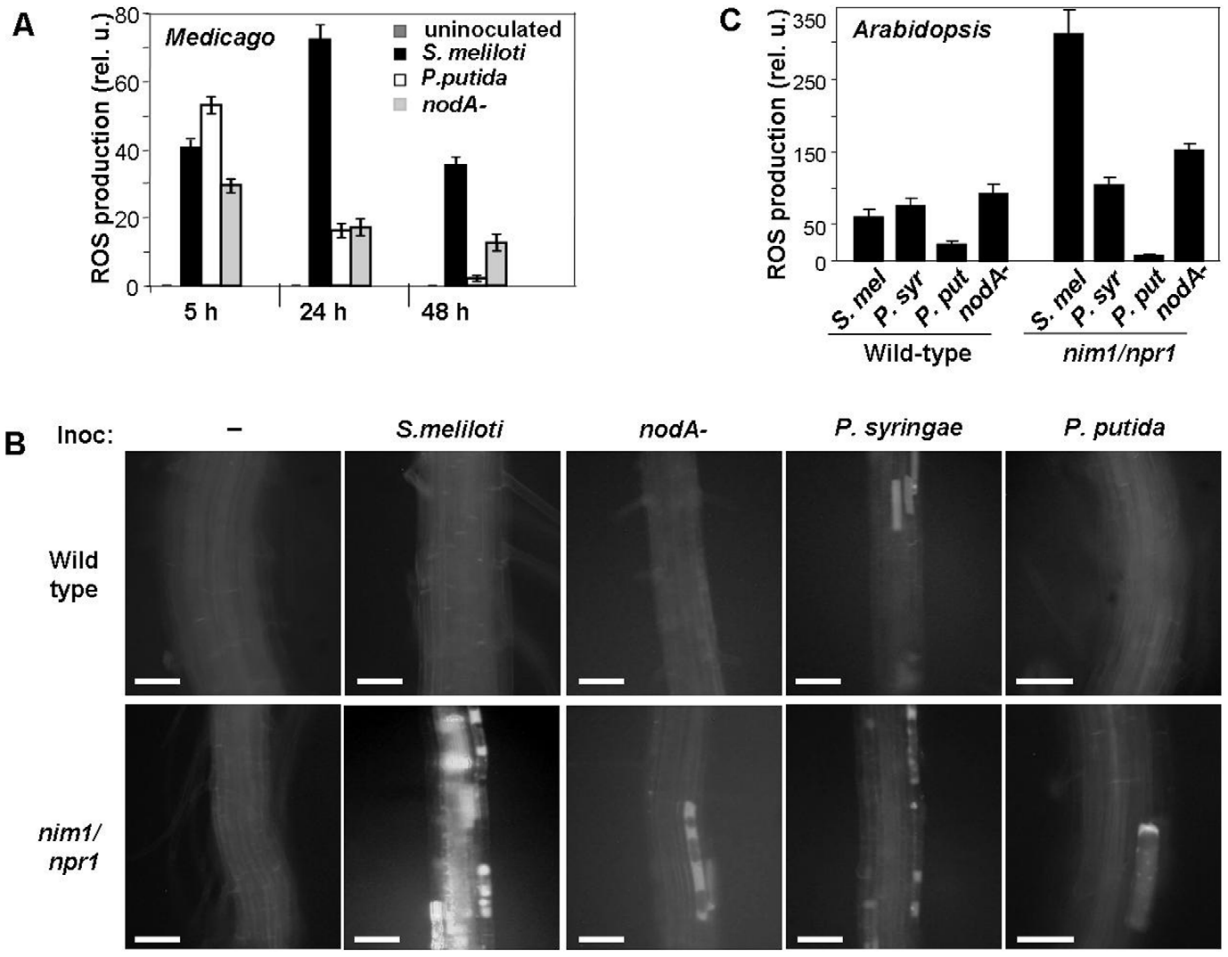
Accumulation of reactive oxygen species (ROS) in roots of Medicago and of wild-type and *nim1/npr1* Arabidopsis seedlings, inoculated with *S. meliloti, P. putida*, or *P. syringae.* (A) Roots of 6 day-old *M. truncatula* seedlings were inoculated with wild-type or *nodA* mutant *S. meliloti*, or with *P. putida*. ROS production was assayed 5, 24 and 48 hours after inoculation by epi-fluorescent microscopy with 2′,7′-dichlorodihydrofluorescein diacetate and narrow-band GFP filter (Ex 485±10 nm/Em 525±10 nm). ROS production in uninoculated roots was below detection level (left bar space in each group). *P. syringae* inoculation produced similar result as *P. putida* (not shown). Error bars indicate standard deviation of the mean (N = 12). (B) Roots of nine day-old wild-type or *nim1/npr1 A. thaliana* seedlings were inoculated with wild-type or *nodA*, or *nodH* (not shown) mutant *S. meliloti*, *P. putida*, or *P. syringae*. ROS production was assayed 24 hours after inoculation by epi-fluorescent microscopy with 2′,7′-dichlorodihydrofluorescein diacetate and a narrow-band GFP filter (Ex 485±10 nm/Em 525±10 nm). All samples were analyzed using identical exposure conditions. Fluorescence from *npr1* plants inoculated with *S. meliloti nodH^−^* mutant was even below uninoculated control (data not shown). Shown are representative *A. thaliana* roots images from four similar experiments 24 hours after inoculation. Bar = 125 µm. (C) Quantitation of ROS production in *A. thaliana* roots, 24 hours after inoculation with wild-type *S. meliloti* (*S.mel*), *nodA* mutants (*nodA^−^*), *P. syringae* (*P.syr*) or *P. putida* (*P. put*). ROS were quantified using ImagePro Plus software package. Error bars indicate standard deviation of the mean (N = 12).

We assayed ROS production in plants inoculated with wild-type or mutants *S. meliloti*, or with *P. putida*, or *P. syringae* bacteria, using 2′,7′-dichlorofluorescin diacetate, which reports ROS production inside the cells [41], [42]. A strong oxidative burst was detected in *M. truncatula* roots already 5 hours after inoculation with either bacterium (Fig. 4A). In plants inoculated with *P. putida* or *P. syringae* bacteria ROS began to decline after the 5 hour peak, and much less ROS were detected after 24 hours, and almost none after 48 hours, particularly in roots inoculated with *P. putida*. However, in plants inoculated with *S. meliloti* the accumulation of ROS peaked after 24 hours, and remained high at least for the first 2 days of interaction (Fig. 4A).

**Figure 5:**
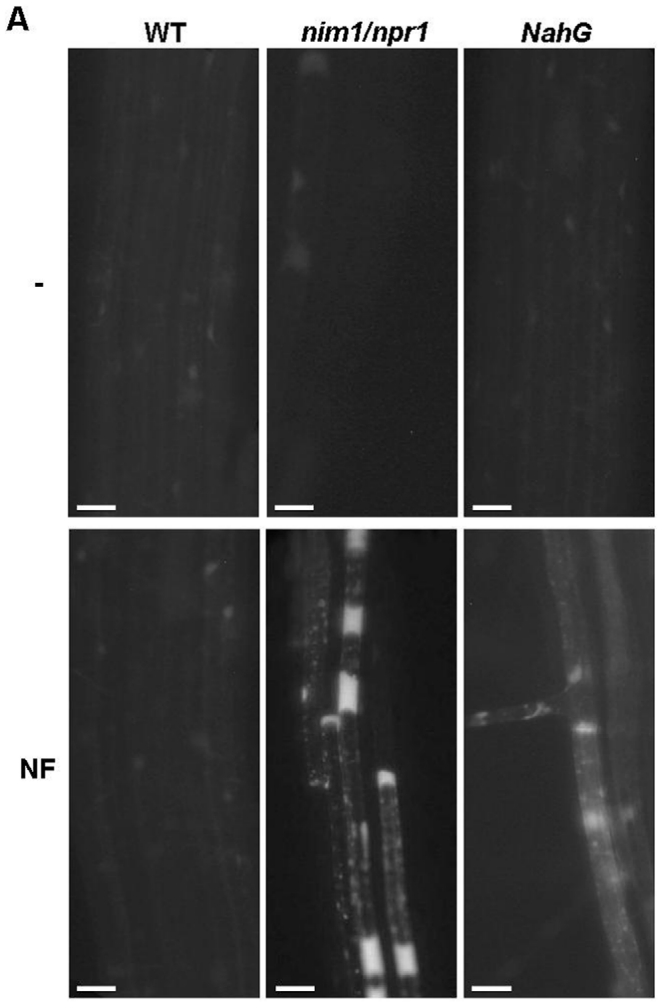
Accumulation of ROS in wild-type, *nim1/npr1*, and *NahG A. thaliana* roots, treated with nod factor. Roots of nine day-old wild-type, *nim1/npr1*, or *NahG* transformants *A. thaliana* seedlings were either left intact or treated with nod factor (NF) and 24 hours later were assayed for ROS production by epi-fluorescent microscopy using 2′,7′-dichlorodihydrofluorescein diacetate and a narrow-band GFP filter (Ex 485±10 nm/Em 525±10 nm). All samples were analyzed using identical exposure conditions. Bars = 50 µm.

To assess the role of intact NF in ROS production, we inoculated *S. meliloti,* mutated in the *nodA* gene, which is required for the synthesis of *N*-acetylglucosamine backbone that is essential for correct NF recognition [43]. The *nodA* mutant rhizobia evoked a considerably smaller ROS response after 5 hours, which was further decreased by 24 hours (Fig. 5A).

In *A. thaliana*, the 24 hour time point post inoculation was selected for all of the experiments, as preliminary tests established it as peak time in ROS production induced by *S. meliloti*. Only negligible amounts of ROS were detected after *S. meliloti* inoculation in the wild-type roots (Fig. 4B, top panel and Fig. 4C). However, a very strong oxidative burst was observed in the *nim1/npr1* mutants inoculated with *S. meliloti* (Fig. 4B, bottom panel and Fig. 4C). To analyze the requirement of intact NF for the recognition of the NF by *A. thaliana*, the *nim1/npr1* mutants were inoculated with *S. meliloti* mutated in *nodA*, which resulted in decreased ROS production, in agreement with the *M. truncatula* data (Fig. 4A). Moreover, strong ROS induction was observed in roots treated with purified wild-type NF, specifically in the *nim1/npr1* mutants or *NahG* transformants (Fig. 5).

**Figure 6:**
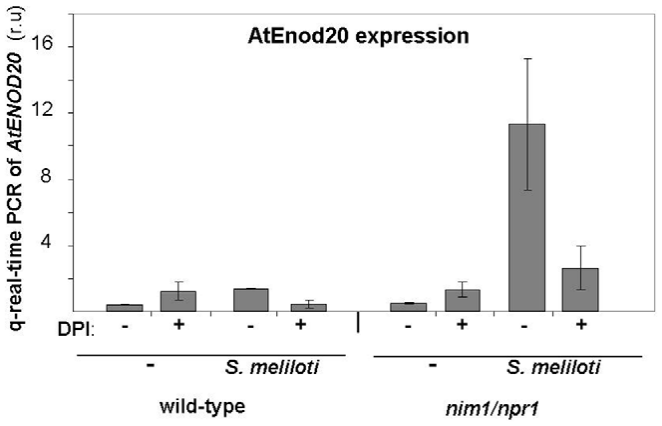
The effect of diphenyleneiodonium on *S.meliloti*-induced ENOD expression. Roots of ten days old wild-type or *nim1/npr1* seedlings were transferred to plates supplemented with 8 µM diphenyleneiodonium (DPI) or replanted on the same (nitrogen poor) medium. After four hours the roots of all plants were inoculated in zone 1 with *S. meliloti*. The expression of *Arabidopsis ENOD20* homolog (At5g57920) was tested two days after inoculation, using quantitative Real-time RT-PCR. The RNA samples were normalized according to *actin*-2 gene expression. The results show the mean of 3 independent repeats for each treatment. Error bars represent SD.

The ROS results are particularly interesting in view of the studies that showed inhibition of the *DMI3* gene (a coordinator of *ENODs* expression) by diphenyleneiodonium (DPI), implicating activation of NADPH oxidase [38], [44]. ROS were also shown to act in the induction of symbiotic peroxidase gene, *RIP1* [39]. We were therefore interested to test the involvement of ROS in expression of *At5g57920*. Ten days-old wild-type and *nim1/npr1* Arabidopsis mutants were pretreated with DPI, as described in [44], after which the plants were inoculated with *S. meliloti*. The expression of *At5g57920* was tested by quantitative real-time RT-PCR four days after inoculation (Fig. 6). DPI suppressed the *At5g57920* transcription, emphasizing the role of ROS in symbiotic interactions.

Our data indicates that ROS induction during the symbiotic interaction is regulated by NF perception. We also show that in legume *M. truncatula* and in non-legume *A. thaliana* the response towards *S. meliloti* is regulated by SA signaling. Suppression of SA signaling, either by decreased SA synthesis, as shown in *M. truncatula* [10] by a yet unknown mechanism, or by mutation of SA-sensing protein, *NPR1* (as shown in *A. thaliana*) brings out similar responses in both plant species. The NPR1 protein may be involved in reducing intracellular ROS, possibly by inducing antioxidants. This suggestion is supported by analysis of NPR1-dependent expression of multiple genes [45], [46]. The intermolecular reduction of the NPR1 protein, which follows the oxidative burst, that results in PR1 induction is in agreement with this suggestion [5], [47].

### The Effect of *NPR1* Overexpression and/or Silencing on *M. truncatula* Root Hair Deformation

*NPR1* is the founding member of a small gene family that contains several NPR1-related or NIM1-like proteins, all of which share the BTB–POZ and the ankyrin-rich repeats domains [48]. To explore the possible role of NPR1 in symbiotic interactions we identified an *NPR1*-like homolog of *M. truncatula* (TC102752) in the public EST database (MtDB2.0). The NPR1-like proteins in *M. truncatula* also form a family (Fig. S6). The *Medicago* gene has 40% identity and 58% similarity to the *A. thaliana* protein and also contains both of the conserved domains that were shown to function in binding and interaction with other proteins, namely the BTB/POZ and ankyrin repeats domains [49]. The *M. truncatula* protein sequence also contains the conserved cysteines that function in the redox-mediated multimerization [5].

**Figure 7:**
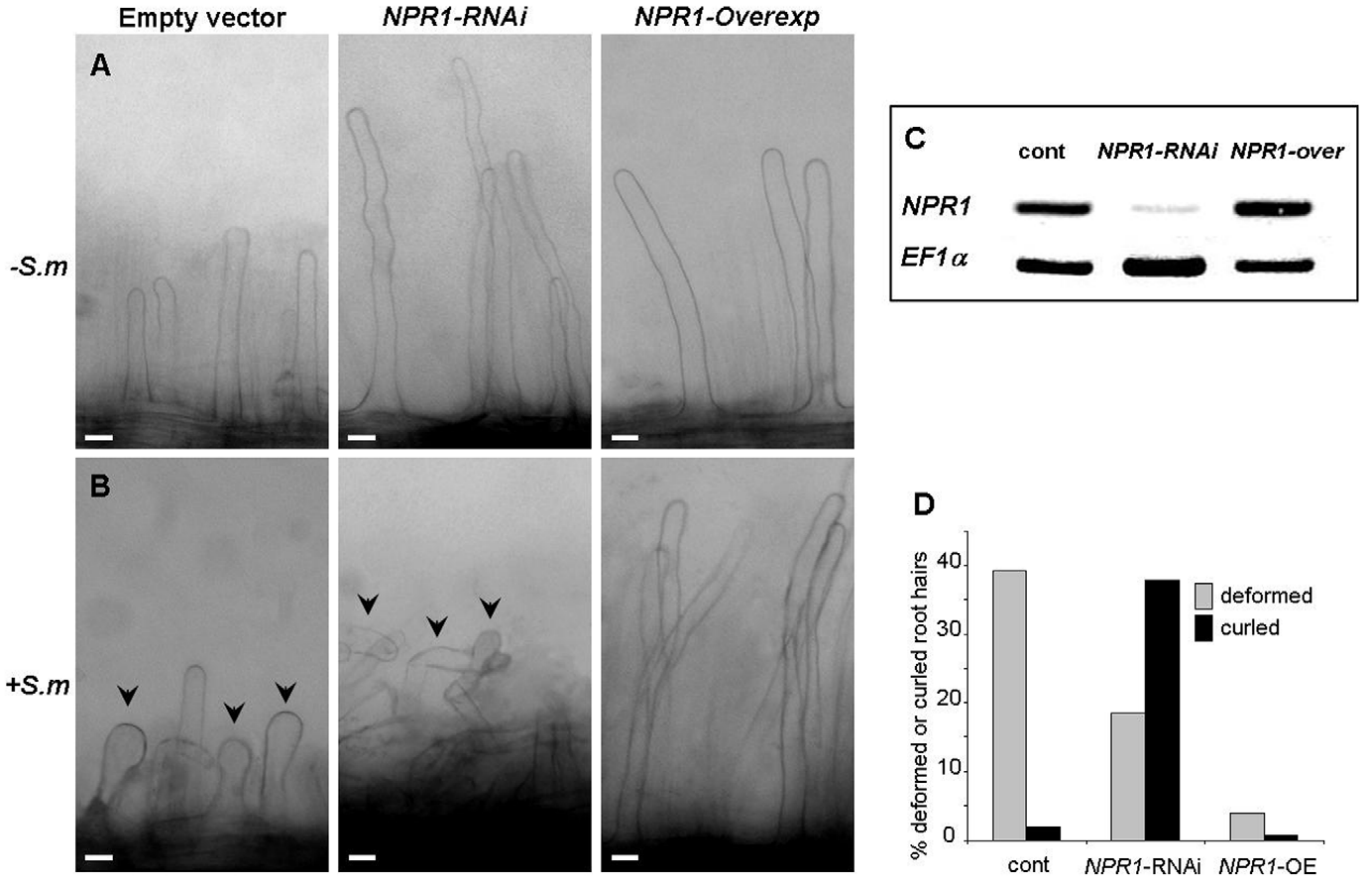
The effect of *NPR1* overexpression or silencing by RNAi on the root hair deformation/curling. (A, B) Roots of seven days-old *M. truncatula* were bombarded with *A. tumefaciens* containing an empty vector, RNAi silencing (*NPR1-RNAi*), or with *NPR1* overexpressing (*NPR1-Overexp*) vectors. After transformation the seedlings were transferred to new plates and either left intact (A), or inoculated with *S. meliloti* after one hour (B). Seedlings were observed for root hair deformation or curling by bright light microscopy two days after inoculation. Roots of six seedlings of each treatment were analyzed. Note the root hair curling already 2 days after *S. meliloti* inoculation in the *NPR1-RNAi* transformed roots, while the empty vector transformed roots (control) show only moderate root hair deformation. Bars = 50 µm. (C) *NPR1* gene expression following transformation, normalized according to *EF1a* gene expression. (D) Quantification of the root hair deformation and curling in *M. truncatula* seedlings overexpressing the *NPR1* gene (*NPR1-*OE) or transformed with RNAi construct (NPR1-RNAi) to suppress *NPR1* gene expression.

To test the role of NPR1 in symbiotic interaction, we bombarded the *M. truncatula* roots with Arabidopsis *NPR1* gene, attached to a constitutive CaMV 35S promoter, in the zone 1 region, using the BIM-LAB-mediated high pressure air-gun apparatus [50]. Such in planta application of *Agrobacterium* vectors has been shown to efficiently deliver the transgenes to different plants, other than Arabidopsis [51]. Expression of the *NPR1* gene was tested two days after the bombardment by RT-PCR, and showed increased expression in transformed roots (Fig. 7C). Plants were analyzed two days after *S. meliloti* inoculation, when root hairs stop elongating and begin to show swelling of the tip [52]. Overexpression of the *NPR1* gene in *M. truncatula* resulted in a strikingly long and straight root hair phenotype (Fig. 7B, compare the *NPR1-Overexp* and empty vector control root hairs; Fig. 7D shows quantification of the above results from 12 seedlings).

To further analyze the role of NPR1 in root hair curling, we silenced the *NPR1* expression in *M. truncatula* roots, prior to *Rhizobium* inoculation, by using the RNAi technique. In order to assure the blocking of interaction between NPR1 and TGA transcription factors, which is essential for induction of PR genes transcription, RNAi was targeted to the NPR1 ankyrin repeats domain that is present in all members of the NPR1-like protein family [53]. Transformation of the *NPR1-RNAi* almost completely blocked the *NPR1* gene expression (Fig. 7C), and resulted in strongly curled root hairs already two days after the *Rhizobium* inoculation (Fig. 7B, compare the *NPR1-RNAi* and empty vector control root hairs). Since normally after two days root hairs show only swelling, and root hair curling occurs around 4 days after *S. meliloti* inoculation [52], this data demonstrate accelerated root hair response in the antisense transformants. These results suggest an inhibitory function of NPR1 on root hair curling.

### Expression of *NPR1*-Dependent Gene Homologs in *M. truncatula*

**Figure 8:**
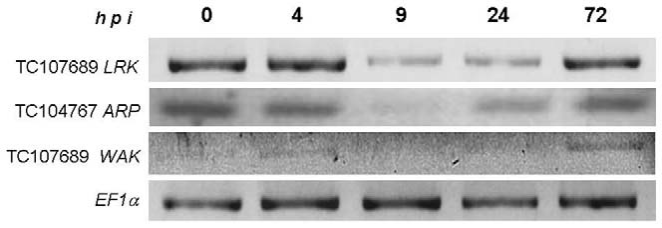
Semiquantitative RT-PCR analysis of pathogenesis-associated gene expression in *M. truncatula*. *M. truncatula* seeds were germinated on an N-free medium and after 6 days inoculated with 10^7^ cells of *S. meliloti*. Total RNA was extracted 24 hours after *Rhizobium* inoculation. The amount of RNA in the samples was normalized according to *EF1a* gene expression. Primers to the *NPR1-*dependent genes in *M. truncatula*: *LRK* (TC107689), *ARP* (TC104767) and *WAK* (TC109689) were identified by BLAST analysis, using the *M. truncatula* homologues to *A. thaliana* gene sequences [59]. *M. truncatula* seedlings were germinated on N-free medium and inoculated with *S. meliloti* three days later. Total RNA was extracted 0, 4, 9, 24, and 72 hours post inoculation (h.p.i.) and analyzed by semiquantitative RT-PCR, using the *M. truncatula EF1a* expression to normalize the amounts of RNA. All experiments were repeated at least three times, independently, with very similar results.

To compare the NPR1-dependent gene expression in *M. truncatula* following *S. meliloti* inoculation with gene expression in *A. thaliana* infected with pathogenic bacteria, we selected several defense genes that were shown to be regulated by NPR1 in *A. thaliana* [45]. The *M. truncatula* orthologs of the Arabidopsis *LRK* (Lectin Receptor Kinase), *ARP* (Ankyrin repeat-containing protein) and *WAK* (Wall Associated Kinase) genes were identified by BLAST analysis of the *M. truncatula* genome project database (http://www.medicago.org/genome/). All of the genes showed constitutive expression in *M. truncatula* roots. However, inoculation of *S. meliloti* caused a window of transcriptional downregulation, starting at 4 hours post inoculation (p.i.) and culminated at 9 hours p.i. (Fig. 8). The gene expression began to recover 24 hours p.i., and resumed to normal levels after 48–72 hours (Fig. 8). These results are in line with the observed reduction in SA accumulation in *M. truncatula* during first 24 hours after *S. meliloti* inoculation [10], [12].

### Concluding Remarks

True symbiotic interactions in plants are thought to be limited to legume family. Our results show that the early basic responses to *Rhizobium* inoculation, such as root hair deformation and induction of early and late nodulin-like genes are conserved between lugume *M. truncatula* and a non-legume *A. thaliana*. However, in *A. thaliana* these responses were observed only in *nim1/npr1* mutant background, suggesting that the NPR1 protein suppresses the plant responses to *Rhizobium*. This suggestion is supported by transient overexpression of the *NPR1* in *M. truncatula* roots, which suppressed the root hair deformation, resulting in straight root hairs (Fig. 7). On the other hand, silencing of the *NPR1* expression by RNAi accelerated the root hair deformation after inoculation of *S. meliloti*. In legumes, the levels of SA are reduced during first days of *Rhizobium* infection, which may result in reduced NPR1-dependent gene expression [10]. In non-legume the symbiotic-like responses were observed only in *nim1/npr1* mutants, or in *NahG* transformants, both of which suppress the SA signaling (Fig. 1). Inhibition of the default SA-mediated defense pathway in legumes during *Rhizobium* infection is probably necessary to allow bacterial entry into the host. Interestingly, inoculation of *S. meliloti* caused a strong oxidative burst in *M. truncatula* and in *A. thaliana nim1/npr1* mutants (Fig. 4, 5), suggesting that the NPR1 protein activates antioxidant responses. It is possible that one or more of the NPR1- dependent genes have antioxidant activity. Alternatively, NF signaling may be less active in the presence of NPR1.

## Materials and Methods

### Biological Material and Plant Treatment

*S. meliloti* and *M. truncatula* were grown as described in [54], except the *M. truncatula* seeds were scarified for 5 min by exposure to concentrated sulfuric acid. The GFP-labeled fluorescent bacteria was a gift from M. Crespi (CNRS, Gif sur Yvette, France). *A. thaliana NPR1* construct was a gift from Xinnian Dong (Duke University, North Carolina). *P. syringae* were grown as described in [55]. Mutant *A. thaliana* seeds were grown on agar plates containing 1/60 MS medium. *S. meliloti* were inoculated on the roots in zone 1 at a concentration of 10^7^ cells. NF was prepared from the *S. meliloti* strain 1021, according to [56].

### Bioinformatics

Phylogenetic N-J tree of the plant genes was constructed by using the Kyoto University ClustalW multiple sequence alignment website, (http://align.genome.jp/). Protein sequences. The *A. thaliana* genes were downloaded from The Arabidopsis Information Resource (TAIR) website (http://www.arabidopsis.org/) and were uploaded to the Kyoto ClustalW website. The *M. truncatula* genes were from public EST database (MtDB2.0).

### RT-PCR Assay

Total RNA was extracted from roots before and after *S. meliloti* inoculation. Roots were frozen in liquid nitrogen. RNA was extracted with Tri Reagent (Molecular Research Center, Inc) and transcribed into cDNA using oligo dT as a primer with SuperScript II reverse transcriptase (Invitrogen). cDNA was amplified by PCR using Taq polymerase and the following primers: *NPR1*: forward TGACTTGTTTTACCTTGAGAA and reverse, AATTATTTTATAGAGAGGAGA. *α-dioxygenase*: forward, GAAGTTTTGGACAAAGTGAGGACT; reverse TGTCAGTTTTAAGAAGCTCCACAG. At5g57920: forward, TAACGAATGGGCTCAAAAGG; reverse CTGGACCGTCGAACTCAGAT. At5g07050: forward, TGGGATTGTGGCATCAAGTA; reverse CCCCTTCCGAGATTTTCATT. *LRK*: forward CAACTCATTTGGTTGGAACTGTAG reverse GGATAAGACAAAGGAAAGTCCTCA. *ARP: forward TCTTCTCCATTTCCTCAATTTCA, reverse TTATTAAGAGCAGCCCACTGAAG.* *WAK*: forward CAGGAGGTTGTCATAAACAAGATG reverse, AAGTGTAACCCGTTGCTAACAAAT. *EF1a* gene, forward TCACATCAACATTGTGGTCATTGGC; reverse, TTGATCTGGTCAAGAGCCTCAAG. *EF1a and Actin2 were used to normalize RNA amounts in M. truncatula and A. thaliana respectively.*

### ROS Production in Plants

Seedlings were taken out of agar plates after 3 days, washed, and transferred to new plates with nitrogen-free medium. ROS in Arabidopsis roots were detected by 10 µm 2′,7′-dichlorofluorescin and ROS levels were quantified with ImagePro Plus analysis package (Media Cybernetics, USA) as described in [38]. Roots were photographed with Nikon Coolpix 4500 camera attached to Olympus IX70 microscope. The fluorescent light pass settings used narrow-band cube (Omega Optical Inc., Brattelboro, VT, USA) 484±20 nm excitation and 535±10 nm emission filters. The pixels of mean density were collected from representative images for statistical analysis (N = 12).

### RNAi Cloning

Silent sites from the Medicago *NPR1* (TC102752) gene were selected, and used to design complementary oligonucleotide primers: forward: 5′- ATCTCTGCCGGAATCAACAC-3′, and reverse: 5′-TCTGATGCACAAGCTCCGTTTTTC-3′. The segment was amplified by PCR and cloned into pENTR using Invitrogen TOPO10-cloning kit and transformed at room temperature for 5 min, then on ice for 30 min, 42°C for 45 sec, and spread on LB solid medium with 50 µg/ml kanamycin. Clones were selected one day later, and sequenced. NPR1- pENTR plasmid was used for the LR recombined reaction using Invitrogen's Gateway LR Clonase II enzyme mix and transformed to TOPO10 competent cells as described above, only the LB contained 100 µg/ml spectomycin and 300 µg/ml streptomycin. Clones were selected one day later, and sequenced using the forward and reverse primers from upstream and downstream sequences of the antisense insertion.

### Transformation of *A. tumefaciens* and Root Bombardment

NPR1-RNAi cloned plasmids were transfected into *A. tumefaciens* GV3101 by freezing in liquid Nitrogen for 5 min and spread on TYNG solid medium containing 50 µg/ml rifampicin, 25 µg/ml Gentamicin, 100 µg/ml spectomycin and 300 µg/ml streptomycin. Monoclonal colonies were selected two days later, and analyzed by PCR for identification. Bacteria were shot with the addition of 1∶1000 M/V carborundum into 7 day-old *M. truncatula* roots, using Bim-LAB apparatus (Bio-Oz, Kibbutz Yad-Mordechai, Israel), essentially, as described in [57]. Plants were taken from plates and the roots were bombarded, using bacterial density of OD600 = 0.5-1 and pressure of 6 Barr, as described in [50]. Plants were then moved to new plates containing N-free medium.

## Supporting Information

Figure S1 Salicylic acid inhibits root hair curling in M. truncatula. (A-F) Three day old M. truncatula seedlings were transferred to plates supplemented with SA (D–F) or replanted on new plates with N-free medium (A–C). The roots of the seedlings were inoculated in zone 1 after 24 h with S. meliloti (C, F) or treated with NF (B, E). SA was applied by dispersing 1 ml of 500 µM SA on top of the plates for 6 days. Pictures were taken 4 days after inoculation, or 2 days after NF treatemnt. At least one hundred root hairs within zone 1 were scored. The percentage of deformed and curled root hairs is indicated on the bottom of each image (upper and lower row, respectively). The ± number indicated the standard deviation. (G) M. truncatula seedlings were germinated on N-free medium and inoculated with S. meliloti three days later. Total RNA was extracted 24 hours after Rhizobium inoculation. The amount of RNA in the samples was normalized according to the EF1a gene expression. The primers to alpha-dioxygenase gene were selected by BLAST analysis using the A. thaliana gene sequences (At3g01420). All experiments were repeated at least three times with very similar results.
(0.78 MB TIF)

Figure S2 The effect of S. meliloti inoculation on A. thaliana dnd1 and nim1/npr1 mutants. A. thaliana seedlings were grown as described in Figure 1. Eight-day old dnd1 or nim1/npr1 mutants were either left untreated (−) or inoculated with S. meliloti (+). Roots were photographed four days after inoculation under bright light. Bar = 25 µm
(0.15 MB TIF)

Figure S3 Treatment of M.truncatula with purified nod factor. Five day old M. truncatula seedlings were exposed to nod factor (+NF) or left intact (−NF). Roots were photographed 3 days after treatment under bright light. Roots of six seedlings of each treatment were analyzed. Bar = 35 µm
(0.10 MB TIF)

Figure S4 Phylogenetic tree of ENOD20. (A) Phylogenetic N-J tree of the Arabidopsis homologs of the M. truncatula early nodulin ENOD20 (TC114239). The tree was constructed using the Kyoto University ClustalW multiple sequence alignment website, (http://align.genome.jp/). Right panel shows the unrooted version of the tree. The genes were chosen using the BLAST program: http://www.arabidopsis.org/wublast/index2.jsp, to the amino acid sequence of TC114239 (ENOD20) at: http://compbio.dfci.harvard.edu/tgi/cgi-bin/tgi/gireport.pl?gudb=medicago (B) Alignment of the Arabidopsis homologs of the M. truncatula ENOD20 (TC114239). The alignment was done using ClustalW2 multiple sequence alignment website from the European Bioinformatics Institute (http://www.ebi.ac.uk/Tools/clustalw2/index.html).
(0.07 MB PDF)

Figure S5 Phylogenetic tree of MtN21. (A) Phylogenetic N-J tree of the Arabidopsis homologs of the M. truncatula MtN21 (TC117774) late nodulin gene. The tree was constructed using the Kyoto University ClustalW multiple sequence alignment website, (http://align.genome.jp/). Right panel shows the unrooted version of the tree. Genes were selected with the BLAST program: http://www.arabidopsis.org/wublast/index2.jsp to the amino acid sequence of TC117774 (MtN21) at: http://compbio.dfci.harvard.edu/tgi/cgi-bin/tgi/gireport.pl?gudb=medicago (B) Alignment of the Arabidopsis homologs of the M. truncatula MtN21 (TC114239) late nodulin gene. The alignment was done using ClustalW2 multiple sequence alignment website from the European Bioinformatics Institute (http://www.ebi.ac.uk/Tools/clustalw2/index.html).
(0.09 MB PDF)

Figure S6 Phylogenetic tree of the NPR1 protein. (A) Phylogenetic N-J tree of the M. truncatula genes that are homologous to the Arabidopsis NPR1. The tree was constructed using the Kyoto University ClustalW multiple sequence alignment website, (http://align.genome.jp/). The right panel shows the unrooted version of the tree. Genes were selected using the BLAST program at http://compbio.dfci.harvard.edu/tgi/cgi-bin/tgi/gireport.pl?gudb=medicago. The proteins were aligned to the At1g64280 (NPR1) available at: http://www.arabidopsis.org/wublast/index2.jsp. (B) Alignment of the M. truncatula homologs of the Arabidopsis NPR1. The alignment was done with ClustalW2 multiple sequence alignment website from the European Bioinformatics Institute (http://www.ebi.ac.uk/Tools/clustalw2/index.html).
(0.04 MB PDF)

Table S1 List of mutants and their putative signaling pathways
(0.06 MB PDF)
