# Supplementary figures and images for: NPR1 Protein Regulates Pathogenic and Symbiotic Interactions between Rhizobium and Legumes and Non-Legumes
Source: PLoS One. 2009 Dec 21;4(12):e8399. doi: 10.1371/journal.pone.0008399 (PMC2793007; doi:10.1371/journal.pone.0008399)

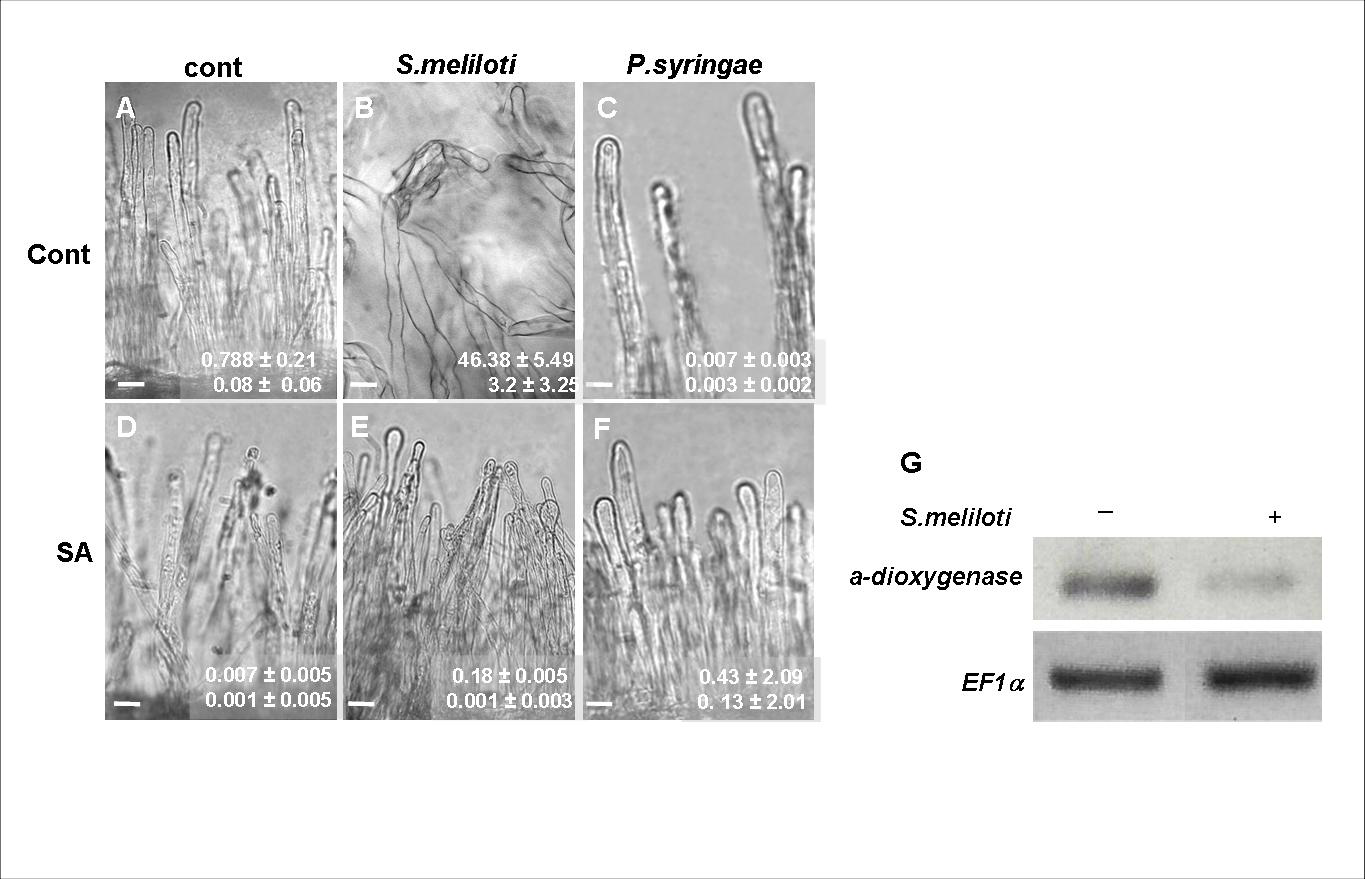

Supplement: Figure S1 — Salicylic acid inhibits root hair curling in M. truncatula. (A-F) Three day old M. truncatula seedlings were transferred to plates supplemented with SA (D–F) or replanted on new plates with N-free medium (A–C). The roots of the seedlings were inoculated in zone 1 after 24 h with S. meliloti (C, F) or treated with NF (B, E). SA was applied by dispersing 1 ml of 500 µM SA on top of the plates for 6 days. Pictures were taken 4 days after inoculation, or 2 days after NF treatemnt. At least one hundred root hairs within zone 1 were scored. The percentage of deformed and curled root hairs is indicated on the bottom of each image (upper and lower row, respectively). The ± number indicated the standard deviation. (G) M. truncatula seedlings were germinated on N-free medium and inoculated with S. meliloti three days later. Total RNA was extracted 24 hours after Rhizobium inoculation. The amount of RNA in the samples was normalized according to the EF1a gene expression. The primers to alpha-dioxygenase gene were selected by BLAST analysis using the A. thaliana gene sequences (At3g01420). All experiments were repeated at least three times with very similar results. (0.78 MB TIF) [file pone.0008399.s001.tif]

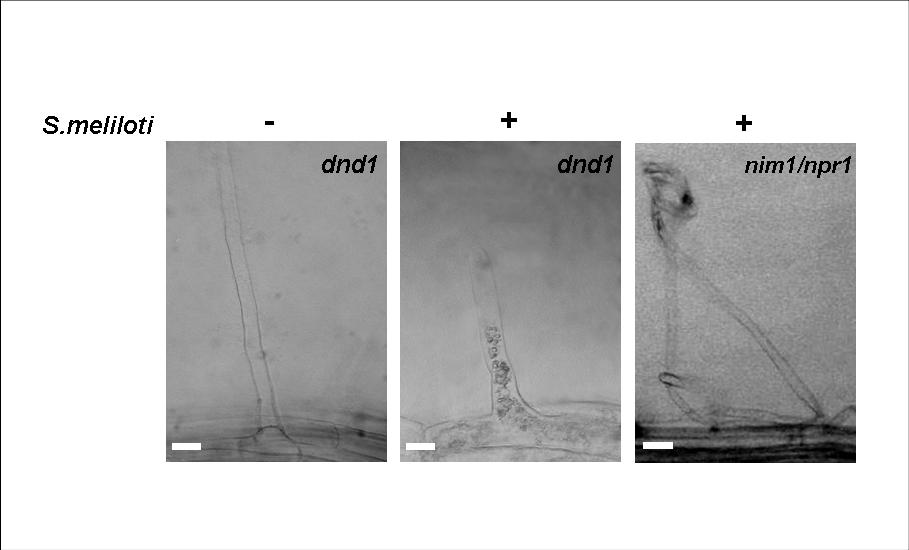

Supplement: Figure S2 — The effect of S. meliloti inoculation on A. thaliana dnd1 and nim1/npr1 mutants. A. thaliana seedlings were grown as described in Figure 1. Eight-day old dnd1 or nim1/npr1 mutants were either left untreated (−) or inoculated with S. meliloti (+). Roots were photographed four days after inoculation under bright light. Bar = 25 µm (0.15 MB TIF) [file pone.0008399.s002.tif]

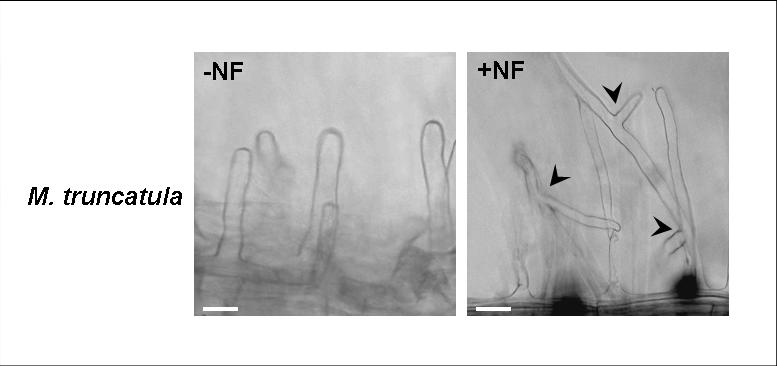

Supplement: Figure S3 — Treatment of M.truncatula with purified nod factor. Five day old M. truncatula seedlings were exposed to nod factor (+NF) or left intact (−NF). Roots were photographed 3 days after treatment under bright light. Roots of six seedlings of each treatment were analyzed. Bar = 35 µm (0.10 MB TIF) [file pone.0008399.s003.tif]

**A**

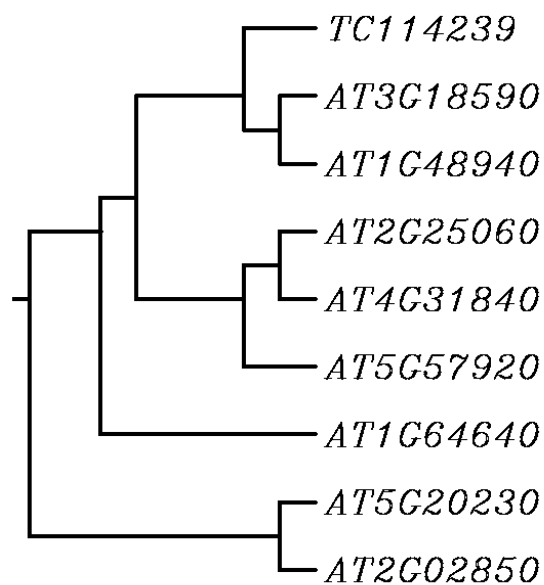

*N-J tree*

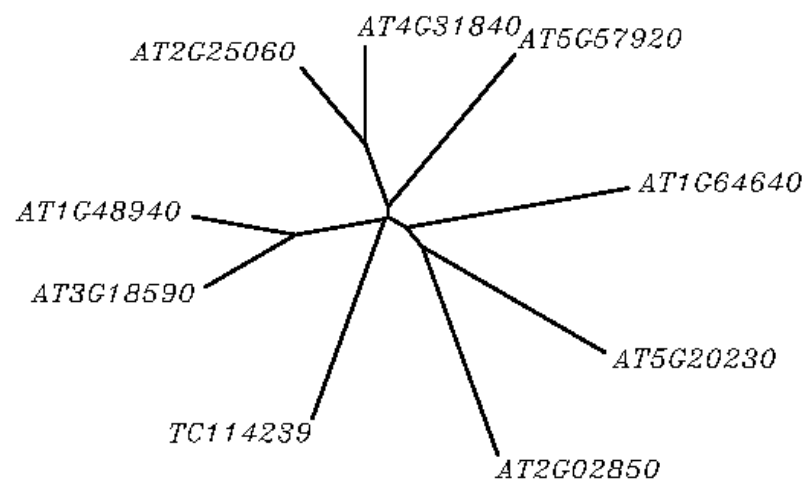

*N-J tree unrooted*

**At5g57920 (*AtENOD20*)**

B

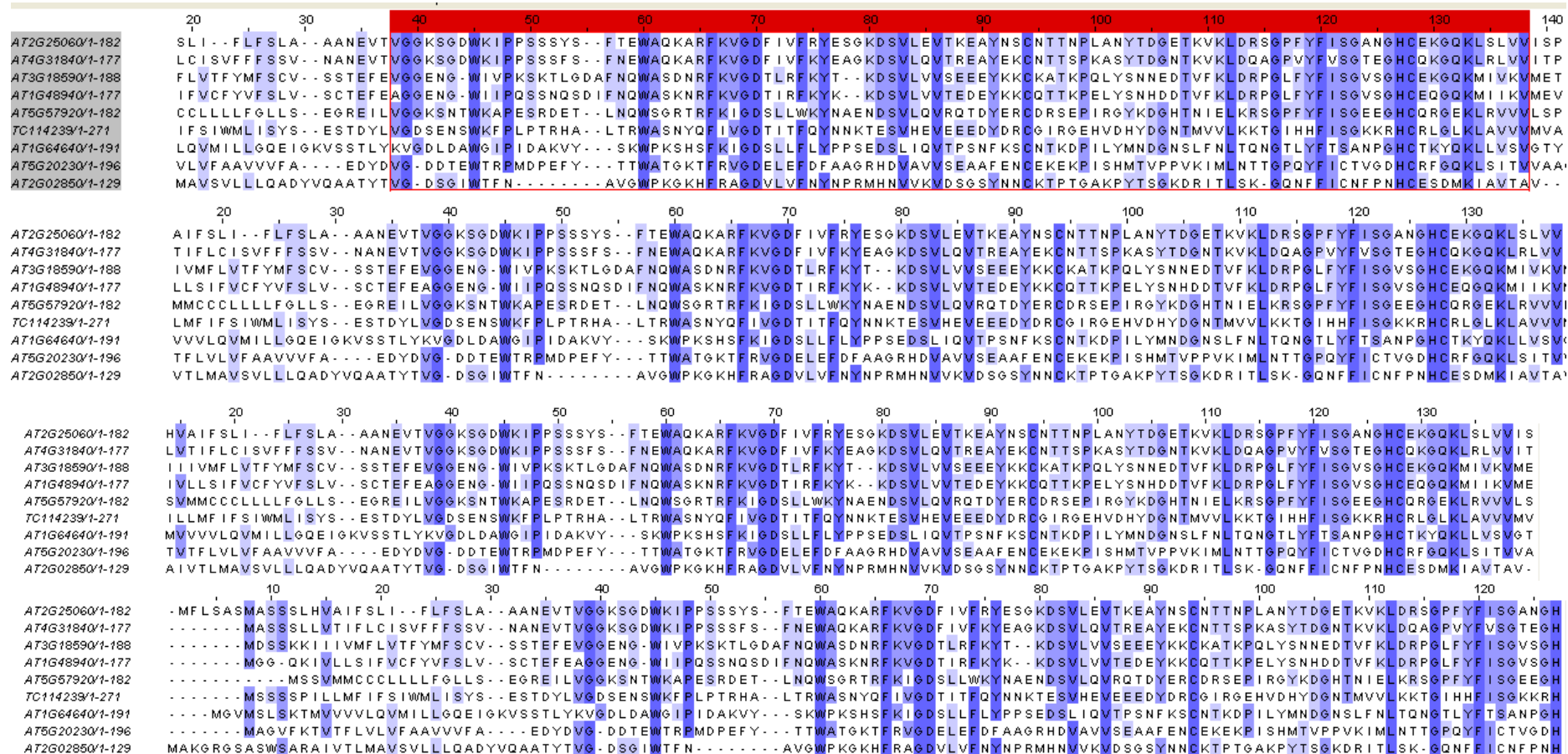

Supplemental Figure S4 B

Supplement: Figure S4 — Phylogenetic tree of ENOD20. (A) Phylogenetic N-J tree of the Arabidopsis homologs of the M. truncatula early nodulin ENOD20 (TC114239). The tree was constructed using the Kyoto University ClustalW multiple sequence alignment website, (http://align.genome.jp/). Right panel shows the unrooted version of the tree. The genes were chosen using the BLAST program: http://www.arabidopsis.org/wublast/index2.jsp, to the amino acid sequence of TC114239 (ENOD20) at: http://compbio.dfci.harvard.edu/tgi/cgi-bin/tgi/gireport.pl?gudb=medicago (B) Alignment of the Arabidopsis homologs of the M. truncatula ENOD20 (TC114239). The alignment was done using ClustalW2 multiple sequence alignment website from the European Bioinformatics Institute (http://www.ebi.ac.uk/Tools/clustalw2/index.html). (0.07 MB PDF) [file pone.0008399.s004.pdf]

**A**

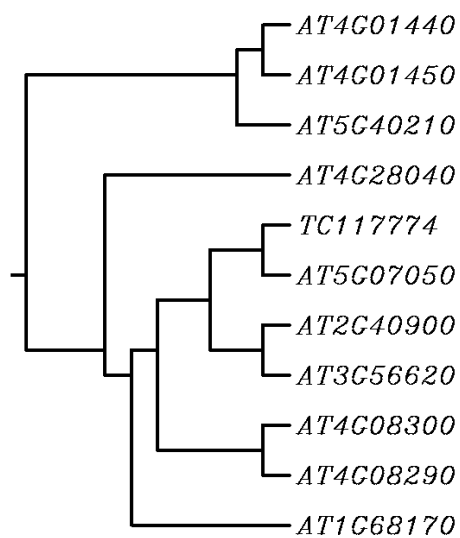

*N-J tree*

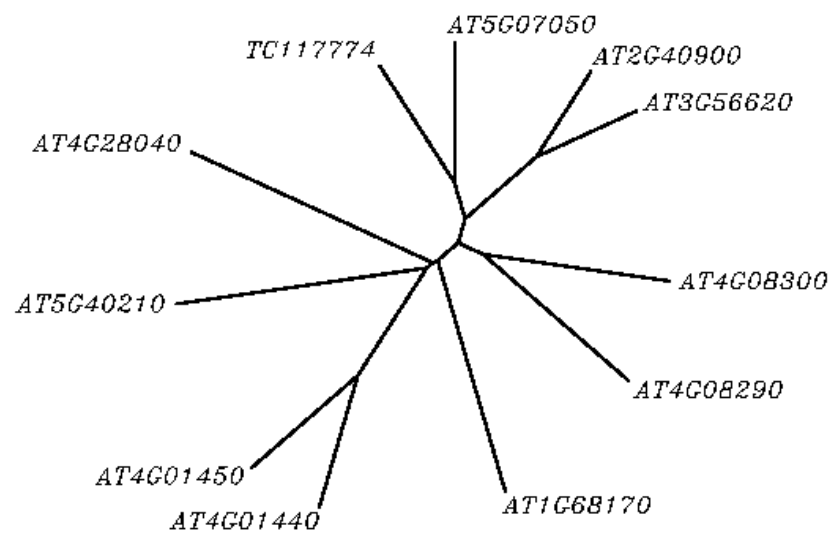

*N-J tree unrooted*

**MtN21**

B

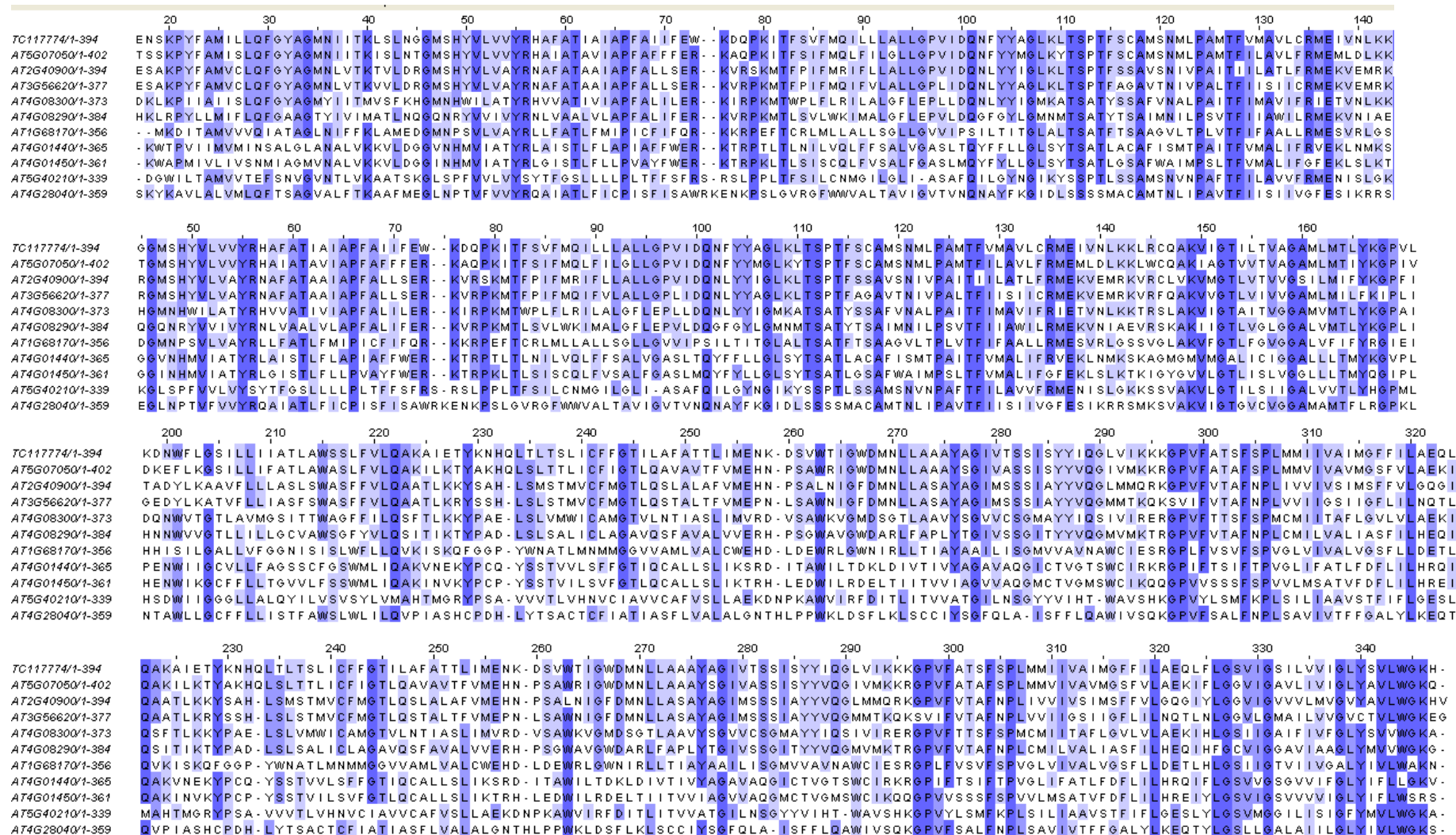

Supplemental Figure S5 B

Supplement: Figure S5 — Phylogenetic tree of MtN21. (A) Phylogenetic N-J tree of the Arabidopsis homologs of the M. truncatula MtN21 (TC117774) late nodulin gene. The tree was constructed using the Kyoto University ClustalW multiple sequence alignment website, (http://align.genome.jp/). Right panel shows the unrooted version of the tree. Genes were selected with the BLAST program: http://www.arabidopsis.org/wublast/index2.jsp to the amino acid sequence of TC117774 (MtN21) at: http://compbio.dfci.harvard.edu/tgi/cgi-bin/tgi/gireport.pl?gudb=medicago (B) Alignment of the Arabidopsis homologs of the M. truncatula MtN21 (TC114239) late nodulin gene. The alignment was done using ClustalW2 multiple sequence alignment website from the European Bioinformatics Institute (http://www.ebi.ac.uk/Tools/clustalw2/index.html). (0.09 MB PDF) [file pone.0008399.s005.pdf]

**A**

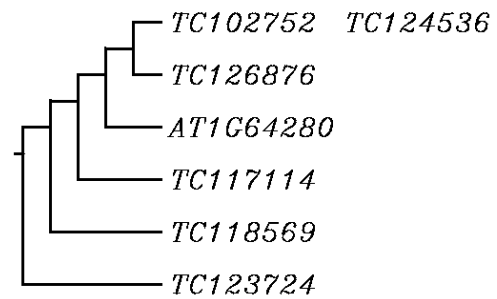

*N-J tree*

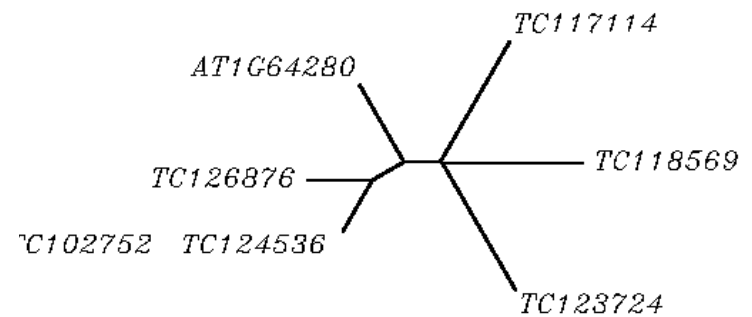

*N-J tree unrooted*

**NPR1**

**B**

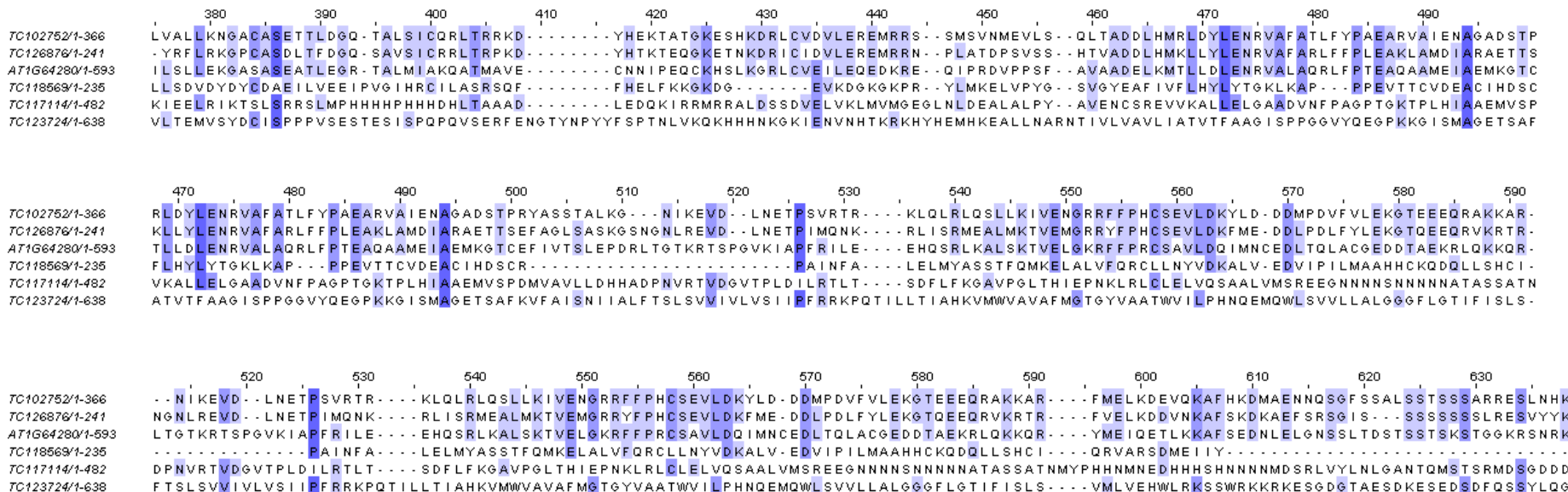

Supplement: Figure S6 — Phylogenetic tree of the NPR1 protein. (A) Phylogenetic N-J tree of the M. truncatula genes that are homologous to the Arabidopsis NPR1. The tree was constructed using the Kyoto University ClustalW multiple sequence alignment website, (http://align.genome.jp/). The right panel shows the unrooted version of the tree. Genes were selected using the BLAST program at http://compbio.dfci.harvard.edu/tgi/cgi-bin/tgi/gireport.pl?gudb=medicago. The proteins were aligned to the At1g64280 (NPR1) available at: http://www.arabidopsis.org/wublast/index2.jsp. (B) Alignment of the M. truncatula homologs of the Arabidopsis NPR1. The alignment was done with ClustalW2 multiple sequence alignment website from the European Bioinformatics Institute (http://www.ebi.ac.uk/Tools/clustalw2/index.html). (0.04 MB PDF) [file pone.0008399.s006.pdf]
