# Supplementary material for: NPR1 Protein Regulates Pathogenic and Symbiotic Interactions between Rhizobium and Legumes and Non-Legumes
Source: PLoS One. 2009 Dec 21;4(12):e8399. doi: 10.1371/journal.pone.0008399 (PMC2793007; doi:10.1371/journal.pone.0008399)
Supplement: Table S1 — List of mutants and their putative signaling pathways (0.06 MB PDF) [file pone.0008399.s007.pdf]

Table I

mutation name	gene name	gene number	gene description	Effect on SA
nim1/npr1	noninducible immunity/ nonexpressor of PR genes	At1g64280	Key regulator of the SA-mediated systemic acquired resistance (SAR) pathway. It is similar to NF kappa B, and contains ankyrin repeats; confers resistance to the pathogens *P. syringae* and *Peronospora parasitica*	insensitive to SA
ndr1	nonrace-specific disease resistance	At3G20600	Required for non-race specific resistance to bacterial and fungal pathogens. Mediates systemic acquired resistance (SAR) response	reduced SA level after infection
dnd1	defense no death 1	At5g15410	A cyclic nucleotide-gated cation channel, conducts K^+ and other cations but excludes Na^+. Conducts Ca^{2+} into cells, involved in the innate immune response to pathogens. Mutants are sensitive to avirulent pathogens.	elevated SA levels
pad4	phytoalexin deficient 4	At3g52430	Encodes a lipase-like gene important for SA signaling and function in resistance gene-mediated and basal plant disease resistance. PAD4 can interact directly with EDS1, another disease resistance signaling proteins.	reduced SA levels
NahG	salicylate hydroxylase	bacterial transgene	decomposes SA to catechol	reduced SA levels
